# Consumer Depletion Alters Seagrass Resistance to an Invasive Macroalga

**DOI:** 10.1371/journal.pone.0115858

**Published:** 2015-02-27

**Authors:** Sarah Caronni, Chiara Calabretti, Maria Anna Delaria, Giuseppe Bernardi, Augusto Navone, Anna Occhipinti-Ambrogi, Pieraugusto Panzalis, Giulia Ceccherelli

**Affiliations:** 1 Department of Earth and Environmental Sciences, University of Pavia, Via S. Epifanio14, I-27100 Pavia, Italy; 2 Department of Science for Nature and Environmental Resources, University of Sassari, Via Piandanna 4, I-07100 Sassari, Italy; 3 Marine Protected Area Tavolara Punta Coda Cavallo, Via Dante 1, I-07026 Olbia (OT), Italy; Texas A&M University at Galveston, UNITED STATES

## Abstract

Few field studies have investigated how changes at one trophic level can affect the invasibility of other trophic levels. We examined the hypothesis that the spread of an introduced alga in disturbed seagrass beds with degraded canopies depends on the depletion of large consumers. We mimicked the degradation of seagrass canopies by clipping shoot density and reducing leaf length, simulating natural and anthropogenic stressors such as fish overgrazing and water quality. *Caulerpa racemosa* was transplanted into each plot and large consumers were excluded from half of them using cages. Potential cage artifacts were assessed by measuring irradiance, scouring by leaf movement, water flow, and sedimentation. Algal invasion of the seagrass bed differed based on the size of consumers. The alga had higher cover and size under the cages, where the seagrass was characterized by reduced shoot density and canopy height. Furthermore, canopy height had a significant effect depending on canopy density. The alteration of seagrass canopies increased the spread of *C. racemosa* only when large consumers were absent. Our results suggest that protecting declining habitats and/or restoring fish populations will limit the expansion of *C. racemosa*. Because MPAs also enhance the abundance and size of fish consuming seagrass they can indirectly promote algal invasion. The effects of MPAs on invasive species are context dependent and require balancing opposing forces, such as the conservation of seagrass canopy structure and the protection of fish grazing the seagrass.

## Introduction

Biotic resistance is the ability of species to reduce the invasion of exotic species. Biotic resistance is particularly well studied in plants, where competition from native plants is commonly assumed to regulate the success of invasion [1,2]. The resistance to invasion is thought to be higher in undisturbed communities [3,4], even though few studies support this relationship [5]. Thus, while habitat structure is considered an important invasion barrier [6,7,8,3,9,10,11], anthropogenic disturbance is considered an invasion promoter [12]. Correlative and manipulative studies have generally found a positive relationship between invasibility and disturbance in terrestrial and marine ecosystems [13,14,15,16,17].

Beyond competition and direct interference among plant species, biotic resistance can arise from any effect of resident species on colonizing invaders, including predation, herbivory and disease. However, few field studies about marine systems have examined the effects of one trophic level on the invasibility of other trophic levels and have highlighted the facilitation of exotics by natives at different trophic levels (*e.g*. [18,19, 20]. Particularly, herbivores may have complex effects on the response of the system: consumers can indirectly facilitate invasions by suppressing primary producers, thus freeing resources [21, 19]. Thus, the effect of herbivores on invasibility can vary from positive to negative, depending on the direction and strength of direct and indirect interactions with native and exotic plants. However, consumers, especially large ones, are more susceptible to local extinction [22,23] while most invasions occur at lower trophic levels [24,25]. Understanding how the reduction of large consumers can affect the structure and function of the ecosystem [26, 27] and influence resident species at trophic levels where invasions are more likely is crucial to improve forecasting invasions and predicting their dynamics.

In marine environments, the deterioration and loss of seagrass beds and the expansion of alternative habitats such as algal turfs or dead rhizomes due to human impacts have been documented in urban areas worldwide [28, 29, 30]. Human activities [31] fragment and reduce the habitat complexity of seagrass meadows and introduced species are often implicated in seagrass declines [32], as invasive macroalgae displace seagrass meadows exposed to human perturbations [33]. However, evidence for their negative effects is largely correlative [34]. In general, species of the genus *Caulerpa* (*C. racemosa* and *C. taxifolia*) are the best-known algal invaders of seagrass beds. With limited experimental evidence, it has been hypothesized that algal invaders respond opportunistically to seagrass degradation [32,34] and that they are passive participants rather than drivers of change [12].

In the Mediterranean Sea, the endemic seagrass *Posidonia oceanica* provides critical shoreline stabilization ecosystem services to coastal systems, but it is experiencing decline in several areas due to habitat destruction and pollution [35]. *Caulerpa racemosa* is spreading in subtidal habitats of the North-Western Mediterranean Sea [36]. Correlative data suggests that degraded *P. oceanica* meadows are more heavily colonised by *C. racemosa* [37, 33, 38]. Katsanevakis et al. found low invasion rates of *C. racemosa* in conserved seagrass meadows, with the macroalga largely restricted to the edges of seagrass meadows or to gaps within them. Experimental evidence indicates that *C. racemosa* growth at the edge of seagrass meadows is regulated by *P. oceanica* shoot density [39] and that its spread is proportional to disturbance intensity. This scenario, however, needs to be understood considering the lack of large consumers driven by the intense exploitation occurring over millennia [40]. MPAs are probably among the few sites where testing consumer interactions and top-down control by large consumers is possible.

A preliminary investigation along the Sardinian coast (Western Mediterranean Sea, Italy) in Tavolara Punta Coda Cavallo MPA was done in 2012 and showed, for the first time, that the introduced alga had a significantly lower abundance at reduced seagrass canopy levels (8.66±1.66, 7.33±1.66, and 13.66±2.33, mean % cover±SE n = 6, at 20%, 50%, and 100% shoot density), illustrating the reverse performance previous researchers had observed [37,33]. Coupled with the high density of fish consumers inhabiting the area [41], it has been hypothesized that consumption of *C. racemosa* at the MPA could occur and be higher in degraded canopies. This is supported by the foraging of several consumers, the sea urchin *Paracentrotus lividus* [42], the fishes *Sarpa salpa* [43, 44], *Spondylosoma cantharus* [45], and *Diplodus sargus* [46].

We tested the general hypothesis that the spread of an introduced alga at disturbed, degraded seagrass canopy sites is dependent on the lack of large consumers. We experimentally mimicked the degradation of *P. oceanica* canopy at the edge of the meadow by clipping shoots simulating natural and anthropogenic stressors such as fish grazing [47] and the effects of poor water quality [28]. *Caulerpa racemosa* was transplanted into each plot and we excluded large fishes from half of them (for all fish species found at the site the minimum size is given in Table 1). Specifically, we tested the hypothesis that the success of the invasive alga *C. racemosa* is greater in disturbed seagrass canopies if consumers are lacking. The experimental design allowed us to test the hypothesis that the spread of *C. racemosa* is determined by the outcome of direct and indirect effects of changes at each trophic level, the seagrass and fish consumers. Although herbivores can lower the abundance of the alga directly by grazing it, indirectly high density of fish herbivores would enhance the susceptibility of *P. oceanica* meadows to invasion of *C. racemosa* by grazing the seagrass canopy, as structured seagrass canopy would protect the understory alga from being grazed.

**Table 1:** Fish occurrence at the study site.

Family	Species	Mean abundance (n = 8)	SE	Length (cm) > mesh size	% escluded by fences	Trophic guild
**Centracanthidae**						
	*Spicara maena*	8.13	8.13	15.0	0	Pla
	*Spicara smaris*	42.24	30.26	19.5	0	Pla
**Labridae**						
	*Coris julis*	140.70	24.55	all lengths	0	Omn
	***Labrus merula***	38.52	38.52	14.5	100.0	Pla
	*Labrus viridis*	7.35	7.35	17.5	0	Pla
	*Symphodus cinereus*	3.06	2.97	14.5	0	Pla
	*Symphodus doderleini*	12.20	4.83	all lengths	0	Pla
	*Symphodus mediterraneus*	7.26	5.37	all lengths	0	Pla
	*Symphodus melanocercus*	6.05	2.35	all lengths	0	Pla
	*Symphodus ocellatus*	18.87	7.25	15.6	0	Pla
	*Symphodus roissali*	3.89	3.89	14.5	0	Pla
	*Symphodus rostratus*	32.99	24.09	16.5	0	Pla
	*Symphodus tinca*	331.89	110.10	14.5	0	Pla
	*Thalassoma pavo*	2.73	2.73	all lengths	0	Omn
**Mullidae**						
	***Mullus surmuletus***	48.23	39.16	18.5	81.8	Car
**Pomacentridae**						
	*Chromis chromis*	52.35	10.69	all lengths	0	Pla
**Serranidae**						
	***Epinephelus marginatus***	16.38	16.38	15.6	100	Pla
	***Serranus cabrilla***	21.33	9.60	16.5	34.2	Pla
	***Serranus scriba***	62.13	21.96	15.0	92.8	Pla
**Sparidae**						
	***Diplodus annularis***	31.41	19.01	11.4	93.5	Omn
	***Diplodus puntazzo***	5.53	5.53	12.0	100.0	Omn
	***Diplodus sargus***	41.40	32.20	11.4	100.0	Omn
	***Diplodus vulgaris***	395.64	255.08	11.4	92.9	Omn
	***Oblada melanura***	58.52	42.13	15.6	86.5	Omn
	***Sarpa salpa***	51.46	51.46	14.5	0	Her
	***Sparus aurata***	7.74	7.74	13.0	100.0	Car
	***Spondyliosoma cantharus***	51.19	35.33	12.0	73.1	Omn
**Tripterygiidae**						
	*Tripterygion melanurus*	0.06	0.06	all lengths	0	Pla

Mean±SE fish biomass (g/50m^2^) estimated at the study site. Minimum fish length (L) per species excludable by cages was calculated by L = FH/PLH where FH = fish height and PLH = proportional length height coefficient. Species in bold can potentially contribute to the differences based on length frequency. Trophic guilds are Her herbivore, Pla Planktotrophic, Car Carnivorous, and Omn Omnivorous.

## Methods

The field experiment was started in June 2013 in a small bay of Tavolara Punta Coda Cavallo MPA (40°35.200 N, 09°48.500 E), in northeast Sardinia (Italy). The authority that issued the permit for the study site was the Director of the MPA.

The field work involved a protected seagrass meadow (*P. oceanica*) that was manipulated by clipping shoots and reducing the canopy height (leaves halved); as this manipulation didn’t involve the meristem the plant recovered within few months [48]. Furthermore, none of the vertebrate species (fishes) involved in the study were collected. They were only indirectly involved as they were excluded by caging experimental units. Very few individuals (n = 5) of *D. sargus* and *S. salpa* (caught within the MPA close to the study area, where both *P*. *oceanica and C*. *racemosa* were present) were bought at the fish market for stomach content analyses. No approval of any animal ethics committee was requested.

### Study system

The protected area includes 15,357 ha and 76 km of coastline with three levels of protection. The study site was located in a B zone where (since 2004) Mediterranean MPA regulations are enforced and fish biomass is significantly correlated with the level of protection [49,50].

At the site, *P. oceanica* canopy structure (5–7 m deep) is well preserved, shoot density is 626.5±1.6 and 456.2±2.3 m^-2^ mean±SE (*n* = 15) in the inner meadow and at the edge, respectively, and with leaf lengths (53.00±2.35 cm, *n* = 30) similar to that in areas without human disturbance [48]. At the site, *C. racemosa* has been spreading since 2009 and it has become common on rocky reefs, on dead *P. oceanica* and at the edge of meadows, while the occurrence of the sea urchin *P*. *lividus* is merely occasional (SC, personal observations).

Fish data were collected at the field site four times in September 2013 [51]. Each time we conducted two 25m×2m transects where the diver swam one way at constant speed, identifying and recording the size of each fish encountered. Fish size was estimated visually and fish recruits (<2 cm total length) were excluded. Fish biomass was estimated from weight-length relationships available in literature and from existing databases [52,53]. The fish assemblage included a total of 28 taxa (Table 1); among these, only *S. salpa* is a major native herbivore. For all species censed in the transects, the portion (percent) biomass excluded by the cages was estimated.

### Treatments

Habitat complexity of the *P. oceanica* bed was manipulated simulating the effects of anthropogenic disturbances and fish grazing: shoot density (100%, 50%, and 20%) was altered by clipping shoots with scissors and canopy height (natural and halved, N and H) by cutting leaves. Treatments were applied to 36 40×40 cm randomly assigned plots on the bed edge, in a completely orthogonal design. Two *C. racemosa* stolons ~ 20 cm in length were transplanted into each plot.

Six replicate plots were established for each shoot density, canopy height combination and within each group three were randomly assigned as large fish herbivore exclusions (F) and the other three as controls (UF). Large fish herbivores were excluded by fencing plots with cages of a plastic covered wire mesh (40×40×70 cm, L×W×H) attached to the substrate with metal corner stakes. Cages also had a transparent nylon net top to protect from leaves. Mesh openings were sufficiently large (5×5 cm) to allow free movement of small fish and invertebrates, but small enough to exclude larger consumers. Visual census data suggested that the biomass excludable from cages was mainly constituted by fishes belonging to *Mullidae*, *Serranidae* and *Sparidae* (Table 1), although only *Diplodus* spp., *S. salpa* and *Spondyliosoma cantharus* likely affected our results.

### Response of *C. racemosa*

Treatment responses were assessed from July to September 2013. The experiment was planned to last longer but prolonged rough seas prevented us from maintaining treatments longer. By sampling at the end of September, however, we estimated the effects of the treatments when *C. racemosa* peaked in abundance. Cover and size of *C. racemosa* in each plot were quantified four times (9^th^ July-T_0_, 29^th^ July-T_1_, 4^th^ September-T_2_, and 29^th^ September 2013-T_3_).

The percent cover of *C. racemosa* in each plot was estimated with a digital camera; on a computer screen a grid of twenty-five sub-quadrats was superimposed onto each image, scoring each sub-quadrat from 0 to 4%. The length of two fronds/plot was measured in the field using callipers.

Each response variable was analysed separately with 3-way ANOVAs (run on the last sampling time data) with herbivory (fenced and unfenced), canopy density (100%, 50% and 20%), and canopy height (natural and halved) as fixed orthogonal factors. Cochran’s test was used to check for homogeneity of variances, while SNK tests were used for *a posteriori* comparisons.

### Procedural controls

Procedural controls were used to detect whether variables, other than herbivore exclusion, were introduced by the cages. For procedural controls, three 40×40 cm plots of *P. oceanica* with 20% shoot density and reduced canopy height were prepared and fenced with cages bearing wide openings at the sides, so that even large herbivores could enter. This is the treatment (*i*.*e*. 20%H) where consumers of algae are predicted to be most active. The cover and size of *C. racemosa* were analysed with a one-way ANOVA (herbivory fenced, procedural control and unfenced) at 20%H canopy treatments (*i*.*e*. where consumers of the alga are mostly active). Cochran’s and SNK tests were run as above.

### Environmental variables

To assess potential caging artifacts and develop a mechanistic understanding of how the seagrass canopy altered the understory species we measured environmental variables. Generally seagrass canopies, including *P. oceanica*, affect the above mentioned species by (1) shading [54,55], (2) scouring [56], (3) lowering water flow [57], (4) increasing sedimentation [58,59], and (5) providing refuge for consumers [60,61]. To provide information on how pruning shoots altered these effects and whether this interacts with caging effects, we measured irradiance, scouring from leaf movement, water flow, and sedimentation at all treatment combinations.

**Irradiance**. Light measurements were taken at 12:00 h on a sunny day using an underwater quantum photometer. Two readings were taken for each of the 6 combinations of density × height, at both F and UF treatments.

**Scour**. Scour was determined using blue, glazed, 100×100 mm tiles painted with a thin coat of white, water-soluble, non-toxic paint. Underwater this paint easily wiped off the tile. On two randomly chosen days with moderate swell, one tile was randomly placed under the canopy in each plot for 24 hrs. The percent cover of blue on each tile (area abraded by *P. oceanica*) was recorded.

**Water flow**. Weighed plaster balls were exposed to water flow for 24 h at the level of *C. racemosa* blades in each plot. On two random days one plaster ball was placed at the center of each plot. Weight loss during field exposure gives time-integrated estimates of water movement. After recovery, balls were rinsed, dried at 60°C for 24 h and weighed. Weight loss was used to estimate flux (cm/s): Flux = 53.65(Me/Mc21), where Me is weight loss during field exposure and Mc is the weight loss of calibration balls.

**Sediment deposition**. On two randomly chosen days sediment traps (150 mm high × 100 mm diameter: aspect ratio > 3) were placed at the center of each plot, with the opening approximately at *C. racemosa* height, for 24 h. Once they had been removed the sediment within each of them was weighed.

Irradiance, scour, water flow, and sediment deposition were analysed with separate 3-way ANOVAs with herbivory (F and UF), canopy density (20%, 50%, and 100%), and canopy height (natural and halved) as fixed orthogonal factors. Cochran’s and SNK tests were run as above.

## Results

### Response of *C. racemosa* to treatments

**Fig 1:**
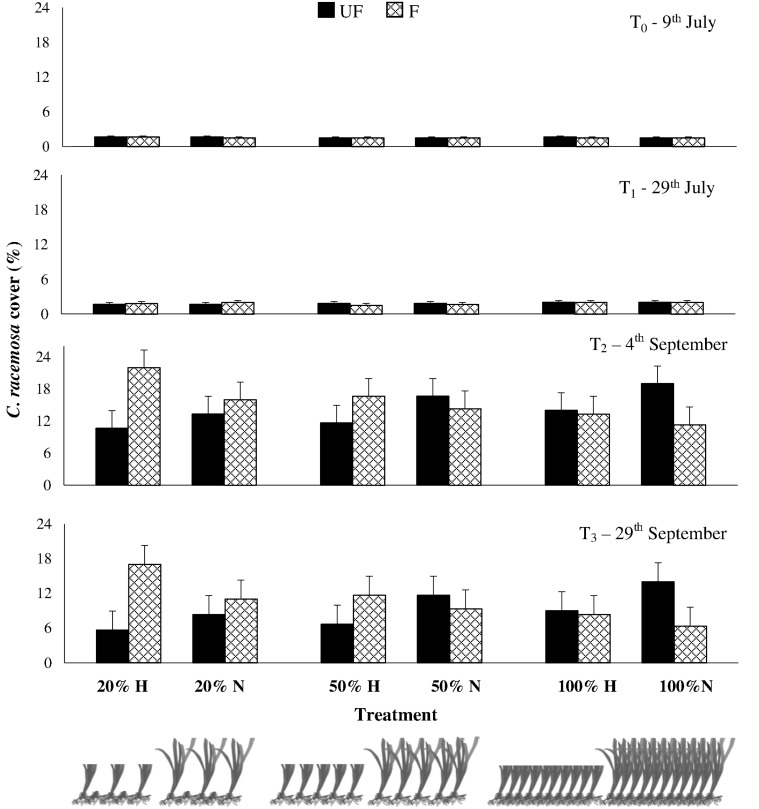
Mean (±SE) *Caulerpa racemosa* percent cover at the four sampling times for the six *Posidonia oceanica* combinations of shoot density (100%, 50%, and 20%) and height (Natural N and halved H) at Fenced (F, checked bars) and Unfenced (UF, black bars) treatments.

**Fig 2:**
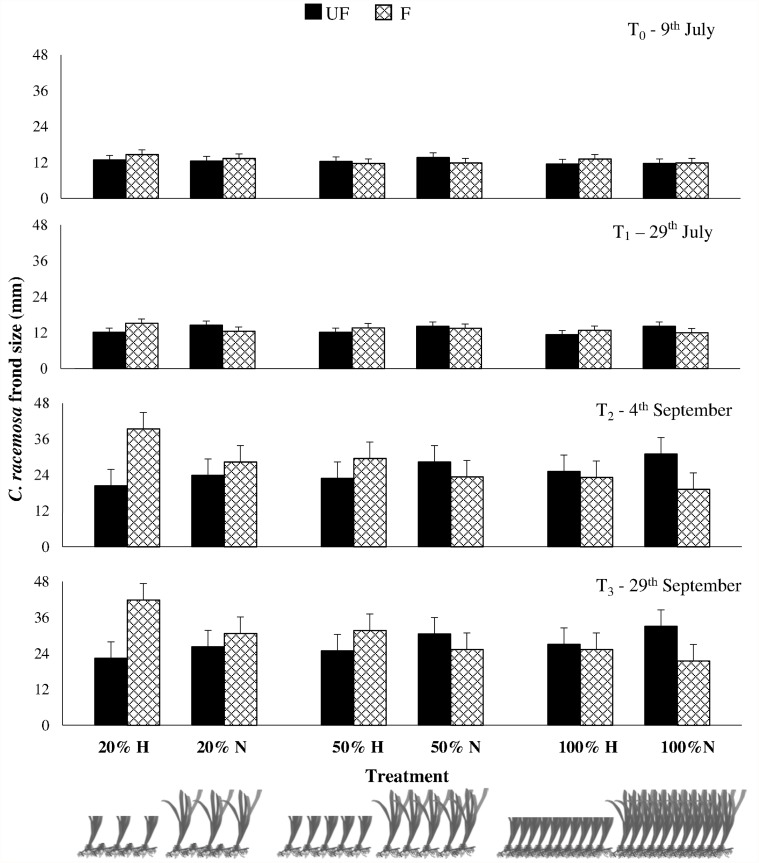
Mean (±SE) *Caulerpa racemosa* frond size at the four sampling times for the six *Posidonia oceanica* combinations of shoot density (100%, 50%, and 20%) and height (Natural N and halved H) at Fenced (F, checked bars) and Unfenced (UF, black bars) treatments.

The two *C. racemosa* fragments transplanted in each plot spread during the course of the experiment and differences in the algal performance among treatments have become evident over time (Figs. 1 and 2; S1 Table). At the end of the experiment, the percent cover and frond size of *C. racemosa* varied consistently across treatments depending on the interactive effects of herbivory, grass height and shoot density (SNK tests in Table 2, Figs. 1 and 2).

**Table 2:** ANOVAS to evaluate *C. racemosa* performance.

ANOVA		*C. racemosa* cover			*C. racemosa* size		
Source	df	MS	F	P	MS	F	P
Herbivore = He	1	17.36	19.53	**0.0002**	39.06	21.15	**0.0001**
Canopy Density = CD	2	3.58	4.03	**0.0309**	38.26	20.71	**0.0000**
Canopy Height = CH	1	1.36	1.53	0.2279	8.51	4.61	**0.0422**
He×CD	2	93.52	105.22	**0.0000**	262.52	142.12	**0.0000**
He×CH	1	132.25	148.78	**0.0000**	339.17	183.61	**0.0000**
CD×CH	2	9.52	10.72	**0.0005**	17.84	9.66	**0.0008**
He×CD×CH	2	0.58	0.66	0.5279	5.05	2.73	0.0852
Residual	24	0.88			1.84		
Cochran’s test		C = 0.2188 ns			C = 0.2293 ns		

SNK test	*C. racemosa* cover	*C. racemosa* size
He×CD		
	SE = 0.38	SE = 0.55
	UF 100%>50%>20%	UF 100%>50%>20%
	F 100%<50%<20%	F 100%<50%<20%
	100% UF>F	100% UF>F
	50% UF = F	50% UF = F
	20% UF<F	20% UF<F
He×CH		
	SE = 0.31	SE = 0.45
	UF N>H	UF N>H
	F N<H	F N<H
	N UF>F	N UF>F
	H UF<F	H UF<F
CD×CH		
	SE = 0.38	SE = 0.55
	100% N<H	100% N<H
	50% N<H	50% N<H
	20% N>H	20% N>H
	N 100% = 50%<20%	N 100% = 50%<20%
	H 100% = 50% = 20%	H 100% = 50% = 20%

Effects of Herbivore (fenced vs unfenced), Canopy Density (100%, 50%, and 20%), and Canopy Height (natural vs. halved) on *C. racemosa* cover and size. SNK tests for comparisons of significant interactions. F = fenced and UF = unfenced refer to the herbivore treatment, while N = natural and H = halved refer to the Canopy height treatment.

In detail, large herbivores exclusion positively affected the alga only at seagrass degradation treatments producing higher algal cover and size where the seagrass canopy was destructured (significant He×CD, Table 2). In fact, a different cage effect was identified depending on *P. oceanica* canopy density: where the seagrass density was unaltered (100%) *C. racemosa* spread was higher in unfenced plots whereas, where the seagrass was deteriorated (20%), algae had larger size and covered a wider part of the plots under the cages. On its turn, the high canopy density of the seagrass positively affected the alga performance where all herbivores were present, as its spread was greater where cages were not present (UF), while the reverse effect of canopy density was found under fencing.

Furthermore, herbivores effects were changed by the deterioration of seagrass canopy density (clipping the shoots) as well as by the deterioration of canopy height (halving leaves length). In fact, where large herbivores were not present (F) the halved canopy led to greater invasion of the alga (significant He×CH, SNK test in Table 2): in other words fencing the halved canopy height had the same effect (*i*.*e*. positive, higher algal cover and size) on the alga of unfencing natural height canopy (Figs. 1 and 2).

Furthermore, seagrass canopy height contributed to *C. racemosa* performance interacting with canopy density effects, irrespectively of the presence of herbivores (significant CD×CH, Table 2). This is evidenced by the lack of effects of seagrass shoot density on the algal performance if *P. oceanica* leaves were halved, while at natural heights the alga invasion was greater at the lowest canopy density (20%).

**Fig 3:**
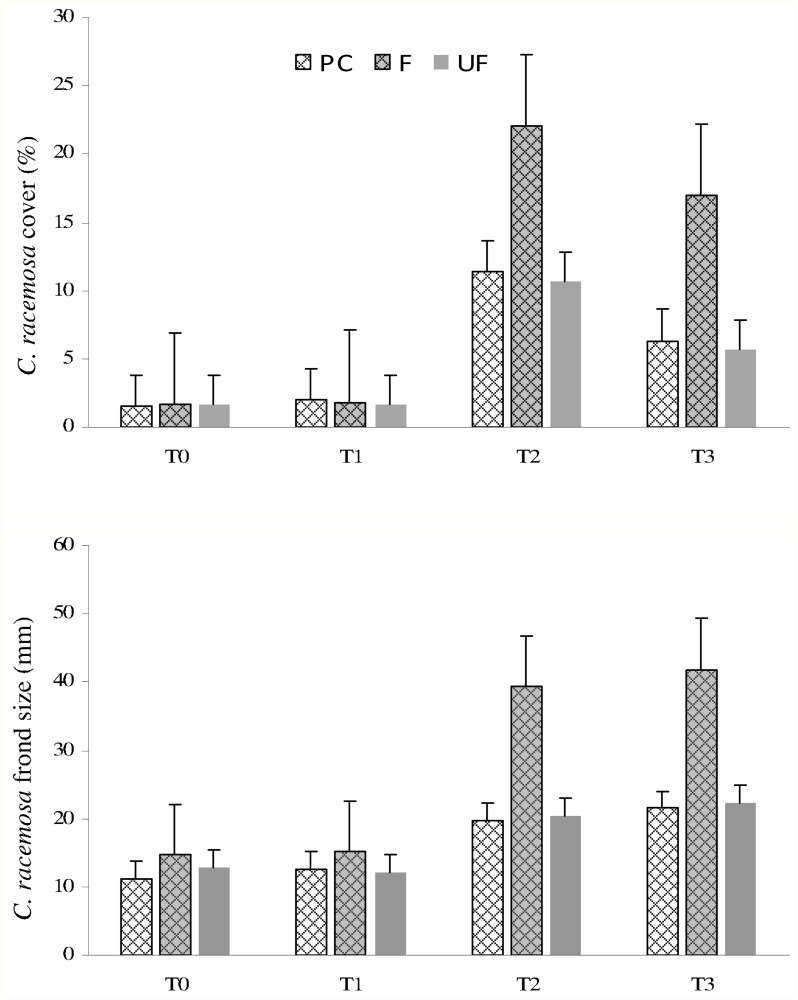
Mean (+SE) *Caulerpa racemosa* percent cover (a) and frond size (b) at the four sampling times for the Procedural control (PC), Fenced (F), and Unfenced (UF) treatments.

During site visits fishes were often found inside the procedural controls and analyses evidenced that no caging artifacts occurred other than excluding fishes; for both response variables open cages (PC) produced effects similar to those of UF and a significantly higher cover and size of the alga were observed in F plots (Table 3, Fig. 3). Furthermore, to clarify about the occurrence of possible consumers of the alga (other than fish), the sea urchin *P*. *lividus* was never found in any of the experimental plots, neither UF nor PC.

**Table 3:** ANOVAs to evaluate procedural artifacts.

ANOVA		*C. racemosa* cover			*C. racemosa* size		
source	df	MS	F	P	MS	F	P
Herbivore = He	2	121.33	218.4	0.0000	407.44	57.3	0.0001
Residual	6	0.55			7.11		
Cochran’s test		C = 0.600 ns			C = 0.6719 ns		
Herbivore							
SNK test		SE = 0.430			SE = 1.539		
		UF = PC<F			UF = PC<F		

Effects of Herbivore (fenced, unfenced, and procedural control) on *C. racemosa* cover and size. SNK tests for comparisons of significant factors. F = fenced, UF = unfenced, and PC = procedural control.

### Seagrass and cage effect on environmental variables

**Fig 4:**
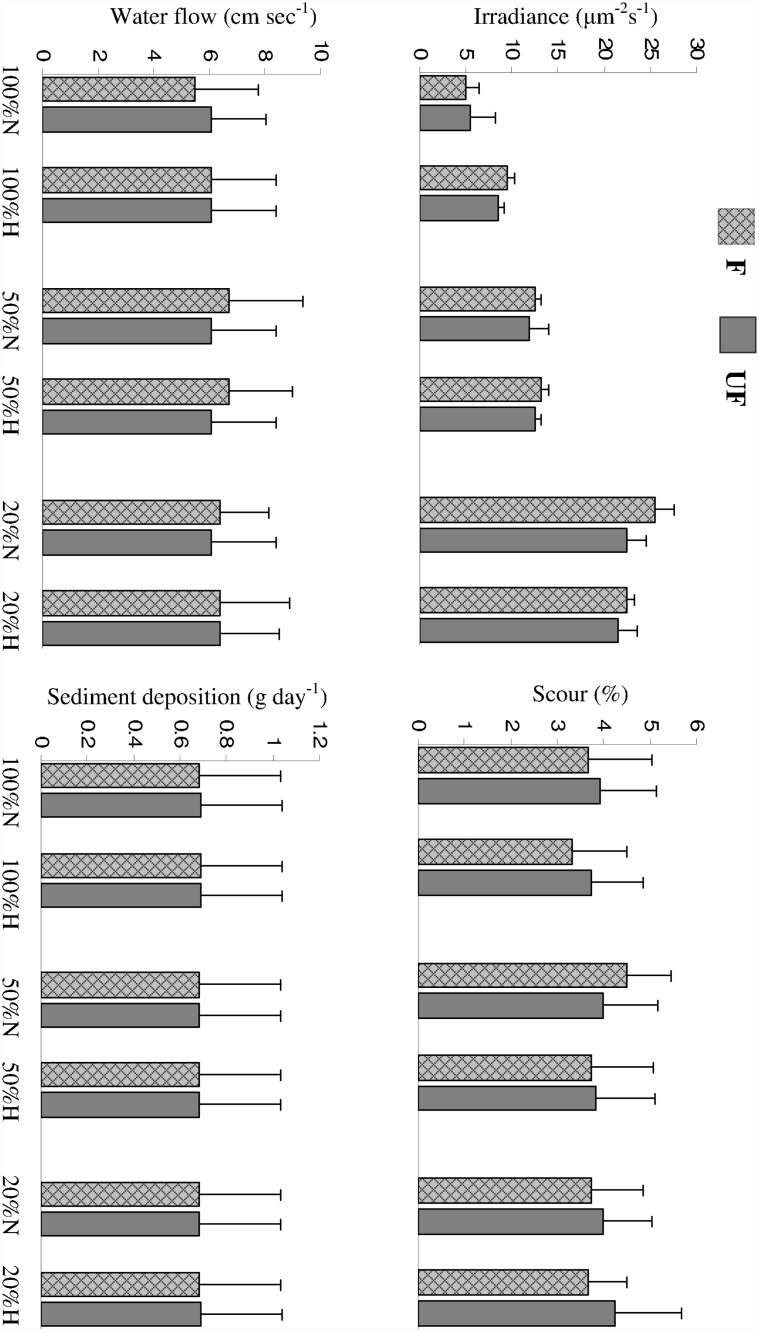
Mean (+SE) irradiance (a), water flow (b), scour (c), and sediment deposition (d) for the six *Posidonia oceanica* combinations of shoot density (100%, 50%, and 20%) and height (Natural N and halved H) at Fenced (F) and unfenced (UF) treatments.

Water flow, scour and sediment deposition were not affected by both fencing and seagrass canopy manipulation (CD and CH, Table 4, Fig. 4; S2 Table); on the contrary, irradiance differed depending on the manipulation of the seagrass canopy, regardless of caging treatment (Table 4). As a matter of fact, we did not detect any effect of fencing on irradiance, which, instead, was highly influenced by *P. oceanica* structure. A significant canopy density×canopy height interaction indicated that light intensity was directly related to the shoot density as significant increases in irradiance were found between 100 and 50%, and between 50 and 20% at both canopy heights (SNK test in Table 4). Higher irradiance was also found at reduced rather than natural height, where shoot density was left unaltered (100%), while at 50 and 20% there were no differences in irradiance between heights (SNK test in Table 4).

**Table 4:** ANOVAs to evaluate environmental variables.

ANOVA		*Irradiance*		*Scour*		*Water flow*		*Sediment deposition*	
source	df	MS	F	MS	F	MS	F	MS	F
Herbivore = He	1	0.0006	2.32	1.25	0.28	0.41	0.03	0.0013	0.00
Canopy Density = CD	2	0.0489	**189.16**	0.46	0.10	1.43	0.09	0.0001	0.00
Canopy Height = CH	1	0.0002	0.58	0.28	0.06	0.41	0.03	0.0001	0.00
He×CD	2	0.0001	0.19	0.23	0.05	1.24	0.08	0.0005	0.00
He×CH	1	0.0001	0.26	0.17	0.04	0.05	0.00	0.0006	0.00
CD×CH	2	0.0032	**12.39**	0.19	0.04	0.14	0.01	0.0007	0.00
He×CD×CH	2	0.0002	0.84	0.02	0.01	0.32	0.02	0.0004	0.00
Residual	12/60	0.0003		4.40		16.16		15205	
Cochran’s test		C = 0. 2348 ns		C = 0.1148 ns		C = 0.1144 ns		C = 0.0859 ns	

SNK test	Canopy Density	Canopy Height	Canopy Height	Canopy Density
Irradiance	100%	Natural<Halved	Natural	100%<50%<20%
	50%	Natural<Halved	Halved	100%<50%<20%
CD×CH	20%	Natural<Halved		
SE = 0.0087				

Effects of Herbivore (fenced vs. unfenced), Canopy Density (100%, 50%, and 20%), and Canopy Height (natural vs. halved) on irradiance, scour, water flow, and sediment deposition. SNK test for comparisons of the significant interaction. Significant (p<0.05) results are in bold. Degrees of freedom of the Residual refer to irradiance/all other analyses.

All the data underlying the manuscript can be found in the archives of Tavolara Punta Coda Cavallo Marine Protected Area and of the Universities of Pavia and Sassari.

## Discussion

Theory predicts that disturbance can promote ecosystem invasion by reducing competition with native species [13,15]. This study provides evidence that different invasion prediction can be made based on the size of the consumers present and that the resistance to invasion is dependent on the conservation status of both competitors and consumers. Alterations to the structure of the seagrass canopies are likely to enhance the spread of *C. racemosa* only when large consumers are absent. This provides indirect evidence that in strongly deteriorated seagrass canopies large consumers can slow the spread of the alga; in unfenced plots the performance of the alga was higher where the seagrass canopy was highly structured. This result is consistent with previous experimental and correlative studies that showed high invasibility of disturbed *P. oceanica* seagrass bed edges by *C. racemosa* as they were from exploited sites where fish assemblages were depleted [39,37,33].

Throughout the Mediterranean Sea, overexploitation has long been recognized as a historical and current pressure that has been leading to major shifts in marine ecosystems [40]. Intense demersal and artisanal fishing has depleted the biomass of consumer species, especially affecting large target species. As a consequence, there is widespread evidence that urbanized areas and protected areas where no surveillance is enforced share the lack of large consumers [49,50]. There is also strong evidence that fishing regulations can re-establish consumer lost interactions and enhance top-down control [62,63]. Thus, in the Mediterranean Sea, enforced MPAs are essentially the only sites where structured fish assemblages are found [50]. Thus, due to consumer depletion, few hotspots of fish diversity and abundance exist. Conversely, in the Mediterranean Sea, there is high variability in the quality of seagrass beds depending on local coastal conditions and historical uses. In urbanized areas the loss of beds (dead) or deteriorated (low density and fragmented patches) *P. oceanica* meadows occur, whereas preserved (high density) meadows can occur in non-urbanized areas [64,65]. This is possible because in shallow water seagrasses can benefit from water clarity, but, because of slow resilience, seagrass meadows can also accumulate impacts related to human activities of terrestrial runoff, sea dumping, nutrient loading, trawling, and anchoring. Moreover, because restrictions can be effective in halting threat-induced trajectories of *P. oceanica* meadows but not in reversing them, MPA seagrass beds are not always healthier than in unprotected areas [65,66].

Human pressures and management have affected to a different extent the competition between *P. oceanica* and *C. racemosa* and the consumers of the latter, suggesting that the effects of biotic resistance to the invasion could be scale dependent [67]. However, the wider effective resistance of the seagrass over consumers indirectly supported by this study (*i*.*e*. no alga can be found inside the healthy seagrass meadow while the occurrence in open habitats is common) emphasizes competition rather than predation, which is also evidenced by current literature with the disproportionate number of studies [2, 20]. The importance of competition in biotic resistance also comes from another classic idea in invasion ecology, the enemies release hypothesis (ERH [68]), which proposes that exotic species escape specialist herbivores in their native range and, therefore, herbivory can have a relatively minor influence on invasive species in their exotic range. This is in contrast with competition, a process invaders do not escape [69]. Although this study does not support ERH, it provides additional evidence that consumers resistance would be possible but *de facto* not frequent: because of the general depletion of the high trophic level, MPAs are probably the only sites where this process can be tested.

Although the dramatic abatement of disturbance in shallow water habitat of a MPA fosters high habitat quality it does not guarantee a barrier against *C. racemosa* invasion. *C. racemosa* is also found where human activities are totally banned as in Tuscan, Apulian, and Sardinian MPAs [33,50]. In these well-preserved systems, probably due to the architectural complexity of the meadows, *C. racemosa* is restricted to the edges of the beds, where rocky bottoms are colonized by turf and erect algae [33].

Although consumers are abundant, the high cover of this alga in MPAs at open habitats suggests a lower contribution of consumers (rather than of seagrasses) to resistance effectiveness [33, 42,50]. Despite this different extent, both *P. oceanica* and consumer assemblages can be alternative forces of resistance to the invasion of C. *racemosa* at seagrass edges. For large scale differences in the conservational status of the two components, the resistance of the seagrass edge is likely a more effective and widespread mechanism rather than predation of large consumers. This is also supported by the lack of *C. racemosa* in the interior of structured meadows and by the presence of the *C. racemosa* in several MPAs at open rocky habitats, where consumers could presumably feed on it. Because no invasions occur in the meadow interiors, the edge of the seagrass meadows is not considered permeable to the invasion of *C. racemosa* because it does not affect the magnitude or the direction of the invasion between open habitats (sand or rocks) and the meadow interiors [70, 71]. The edge of seagrass meadows are important structural features but not functional components of it, unlike what is suggested for forest edges and landscapes [71].

Mechanisms responsible for *C. racemosa* not invading the interior of healthy meadows have been neglected. Irradiance is the physical variable most highly correlated to the architecture of the seagrass canopy and likely responsible for the higher performance of the alga where resident *P. oceanica* had been depleted and large herbivores were not present (20%HF). Low irradiance inside the meadow with high canopy structure cannot be responsible for the initial failure of *C. racemosa* invasion, as shading inside the bed and just a few tens of centimetres from the edge habitats must be similar, and second because the alga is supposed to be shade tolerant for the high performance in deep waters [72,73]. This is in contrast with terrestrial processes as shade tolerant plants are known to invade intact forests from the edges [5]. Thus, mechanisms other than irradiance prevent the penetration into the meadow.

The removal of large consumers, irrespective of species, had clear positive effects on the length of fronds and the penetration of stolons at depleted seagrass edge, suggesting that the competitive interaction between the seagrass and the seaweed may decrease in the presence of predation [74]. Although at the site several fish species were fenced off because of their size, only few have to be considered responsible for the differences, as many of them are known to be planktotrophic or carnivorous. Specifically, *S. salpa* (herbivore) and *Diplodus* species (omnivore, although their relative occurrence is very different) are those thought to have affected the results on *C. racemosa* [46,75]. This is corroborated by stomach content analysis of *D. sargus* (TL 21.96±0.81, mean ± SE) and *S. salpa* (22.87±0.77) that revealed that they all had fed on *C. racemosa* (31.96% ± 2.33 and 41.18% ± 0.44 of wet biomass, respectively). Therefore, besides the direct negative effects on *C. racemosa*, *S. salpa* could also have indirect positive effects on the understory alga, as it can affect the seagrass habitat through the consumption of leaves, modifying the structure of the canopy [76,61,21]. Therefore, these consumers may regulate the spread of the exotic clonal alga by influencing their abundance directly, and by triggering morphology that confers higher invasibility.

Our findings suggest that conservation strategies to protect declining habitats and to restore overexploited fish populations (*i*.*e*. MPAs) may indirectly promote resistance to the expansion of *C. racemosa*. However, because MPAs can also enhance the abundance and size of fishes preying on seagrass leaves (*i*.*e*. *S. salpa*) they can contribute to the reduction in *P. oceanica* canopy cover, promoting the invasion of the alga. Therefore, although maintaining the resistance to invasion should be among the ecosystem services provided by seagrass beds [77], the overall effects of MPAs on the invasive species will be context dependent, due to a balance between contrasting forces, and that strategies for controlling the establishment and spread of invasive species limited to the management of anthropogenic perturbations may be inadequate under some circumstances.

## Supporting Information

S1 Table Raw data regarding *C. racemosa* growth. *C. racemosa* percent cover and frond size in each unit (treatment abbreviations considering: shoot density (100%, 50%, and 20%) and height (Natural N and halved H) in Fenced (F) and unfenced (UF) units) during the four sampling conducted in the study period. C: control units.
(DOCX)

S2 Table Raw data regarding the considered environmental variables. Scouring from leaf movement (Scour), water flow, and sedimentation (Sediment) values at all treatment combinations (treatment abbreviations considering: shoot density (100%, 50%, and 20%) and height (Natural N and halved H) in Fenced (F) and unfenced (UF) units). C: control units.
(DOCX)
