# Supplementary material for: Consumer Depletion Alters Seagrass Resistance to an Invasive Macroalga
Source: PLoS One. 2015 Feb 27;10(2):e0115858. doi: 10.1371/journal.pone.0115858 (PMC4344340; doi:10.1371/journal.pone.0115858)
Supplement: S1 Table — C. racemosa percent cover and frond size in each unit (treatment abbreviations considering: shoot density (100%, 50%, and 20%) and height (Natural N and halved H) in Fenced (F) and unfenced (UF) units) during the four sampling conducted in the study period. C: control units. (DOCX) [file pone.0115858.s001.docx]

| **C. RACEMOSA GROWTH** | | | | | |
| --- | --- | --- | --- | --- | --- |
| **Date** | **Time** | **Unit** | **Treatment** | **Length (mm)** | **Cover (%)** |
| 9 July | T_0_ | 25 | H100Ufa | 25 | 2 |
| 9 July | T_0_ | 11 | H100Ufb | 11 | 1,5 |
| 9 July | T_0_ | 4 | H100Ufc | 4 | 1,5 |
| 9 July | T_0_ | 9 | H50Ufa | 9 | 1,5 |
| 9 July | T_0_ | 30 | H50Ufb | 30 | 1,5 |
| 9 July | T_0_ | 12 | H50Ufc | 12 | 1,5 |
| 9 July | T_0_ | 20 | H20Ufa | 20 | 1,5 |
| 9 July | T_0_ | 36 | H20Ufb | 36 | 1,5 |
| 9 July | T_0_ | 21 | H20Ufc | 21 | 2 |
| 9 July | T_0_ | 5 | H100Fa | 5 | 1,5 |
| 9 July | T_0_ | 13 | H100Fb | 13 | 1,5 |
| 9 July | T_0_ | 32 | H100Fc | 32 | 1,5 |
| 9 July | T_0_ | 2 | H50Fa | 2 | 1,5 |
| 9 July | T_0_ | 6 | H50Fb | 6 | 1,5 |
| 9 July | T_0_ | 16 | H50Fc | 16 | 1,5 |
| 9 July | T_0_ | 33 | H20Fa | 33 | 1,5 |
| 9 July | T_0_ | 22 | H20Fb | 22 | 1,5 |
| 9 July | T_0_ | 15 | H20Fc | 15 | 2 |
| 9 July | T_0_ | 37 | N100Ufa | 37 | 1,5 |
| 9 July | T_0_ | 17 | N100Ufb | 17 | 1,5 |
| 9 July | T_0_ | 1 | N100Ufc | 1 | 1,5 |
| 9 July | T_0_ | 14 | N50Ufa | 14 | 1,5 |
| 9 July | T_0_ | 38 | N50Ufb | 38 | 1,5 |
| 9 July | T_0_ | 35 | N50Ufc | 35 | 1,5 |
| 9 July | T_0_ | 24 | N20Ufa | 24 | 1,5 |
| 9 July | T_0_ | 31 | N20Ufb | 31 | 2 |
| 9 July | T_0_ | 19 | N20Ufc | 19 | 1,5 |
| 9 July | T_0_ | 3 | N100Fa | 3 | 1,5 |
| 9 July | T_0_ | 39 | N100Fb | 39 | 1,5 |
| 9 July | T_0_ | 28 | N100Fc | 28 | 1,5 |
| 9 July | T_0_ | 7 | N50Fa | 7 | 1,5 |
| 9 July | T_0_ | 18 | N50Fb | 18 | 1,5 |
| 9 July | T_0_ | 29 | N50Fc | 29 | 1,5 |
| 9 July | T_0_ | 27 | N20Fa | 27 | 1,5 |
| 9 July | T_0_ | 8 | N20Fb | 8 | 1,5 |
| 9 July | T_0_ | 10 | N20Fc | 10 | 1,5 |
| 9 July | T_0_ | 23 | C1 | 23 | 1,5 |
| 9 July | T_0_ | 26 | C2 | 26 | 1,5 |
| 9 July | T_0_ | 34 | C3 | 34 | 1,5 |
| 29 July | T_1_ | 25 | H100Ufa | 25 | 2 |
| 29 July | T_1_ | 11 | H100Ufb | 11 | 2 |
| 29 July | T_1_ | 4 | H100Ufc | 4 | 2 |
| 29 July | T_1_ | 9 | H50Ufa | 9 | 2 |
| 29 July | T_1_ | 30 | H50Ufb | 30 | 2 |
| 29 July | T_1_ | 12 | H50Ufc | 12 | 1,5 |
| 29 July | T_1_ | 20 | H20Ufa | 20 | 1,5 |
| 29 July | T_1_ | 36 | H20Ufb | 36 | 1,5 |
| 29 July | T_1_ | 21 | H20Ufc | 21 | 2 |
| 29 July | T_1_ | 5 | H100Fa | 5 | 2 |
| 29 July | T_1_ | 13 | H100Fb | 13 | 2 |
| 29 July | T_1_ | 32 | H100Fc | 32 | 2 |
| 29 July | T_1_ | 2 | H50Fa | 2 | 1,5 |
| 29 July | T_1_ | 6 | H50Fb | 6 | 1,5 |
| 29 July | T_1_ | 16 | H50Fc | 16 | 1,5 |
| 29 July | T_1_ | 33 | H20Fa | 33 | 1,5 |
| 29 July | T_1_ | 22 | H20Fb | 22 | 1,5 |
| 29 July | T_1_ | 15 | H20Fc | 15 | 2,5 |
| 29 July | T_1_ | 37 | N100Ufa | 37 | 1,5 |
| 29 July | T_1_ | 17 | N100Ufb | 17 | 2 |
| 29 July | T_1_ | 1 | N100Ufc | 1 | 2,5 |
| 29 July | T_1_ | 14 | N50Ufa | 14 | 2 |
| 29 July | T_1_ | 38 | N50Ufb | 38 | 1,5 |
| 29 July | T_1_ | 35 | N50Ufc | 35 | 2 |
| 29 July | T_1_ | 24 | N20Ufa | 24 | 1,5 |
| 29 July | T_1_ | 31 | N20Ufb | 31 | 2 |
| 29 July | T_1_ | 19 | N20Ufc | 19 | 1,5 |
| 29 July | T_1_ | 3 | N100Fa | 3 | 2 |
| 29 July | T_1_ | 39 | N100Fb | 39 | 1,5 |
| 29 July | T_1_ | 28 | N100Fc | 28 | 2,5 |
| 29 July | T_1_ | 7 | N50Fa | 7 | 2 |
| 29 July | T_1_ | 18 | N50Fb | 18 | 1,5 |
| 29 July | T_1_ | 29 | N50Fc | 29 | 1,5 |
| 29 July | T_1_ | 27 | N20Fa | 27 | 2 |
| 29 July | T_1_ | 8 | N20Fb | 8 | 2 |
| 29 July | T_1_ | 10 | N20Fc | 10 | 2 |
| 29 July | T_1_ | 23 | C1 | 23 | 2 |
| 29 July | T_1_ | 26 | C2 | 26 | 2,5 |
| 29 July | T_1_ | 34 | C3 | 34 | 1,5 |
| 4 Septmber | T_2_ | 25 | H100Ufa | 25 | 14 |
| 4 Septmber | T_2_ | 11 | H100Ufb | 11 | 15 |
| 4 Septmber | T_2_ | 4 | H100Ufc | 4 | 13 |
| 4 Septmber | T_2_ | 9 | H50Ufa | 9 | 12 |
| 4 Septmber | T_2_ | 30 | H50Ufb | 30 | 11 |
| 4 Septmber | T_2_ | 12 | H50Ufc | 12 | 12 |
| 4 Septmber | T_2_ | 20 | H20Ufa | 20 | 10 |
| 4 Septmber | T_2_ | 36 | H20Ufb | 36 | 11 |
| 4 Septmber | T_2_ | 21 | H20Ufc | 21 | 11 |
| 4 Septmber | T_2_ | 5 | H100Fa | 5 | 15 |
| 4 Septmber | T_2_ | 13 | H100Fb | 13 | 12 |
| 4 Septmber | T_2_ | 32 | H100Fc | 32 | 13 |
| 4 Septmber | T_2_ | 2 | H50Fa | 2 | 17 |
| 4 Septmber | T_2_ | 6 | H50Fb | 6 | 16 |
| 4 Septmber | T_2_ | 16 | H50Fc | 16 | 17 |
| 4 Septmber | T_2_ | 33 | H20Fa | 33 | 21 |
| 4 Septmber | T_2_ | 22 | H20Fb | 22 | 23 |
| 4 Septmber | T_2_ | 15 | H20Fc | 15 | 22 |
| 4 Septmber | T_2_ | 37 | N100Ufa | 37 | 18 |
| 4 Septmber | T_2_ | 17 | N100Ufb | 17 | 20 |
| 4 Septmber | T_2_ | 1 | N100Ufc | 1 | 19 |
| 4 Septmber | T_2_ | 14 | N50Ufa | 14 | 16 |
| 4 Septmber | T_2_ | 38 | N50Ufb | 38 | 17 |
| 4 Septmber | T_2_ | 35 | N50Ufc | 35 | 17 |
| 4 Septmber | T_2_ | 24 | N20Ufa | 24 | 15 |
| 4 Septmber | T_2_ | 31 | N20Ufb | 31 | 12 |
| 4 Septmber | T_2_ | 19 | N20Ufc | 19 | 13 |
| 4 Septmber | T_2_ | 3 | N100Fa | 3 | 11 |
| 4 Septmber | T_2_ | 39 | N100Fb | 39 | 12 |
| 4 Septmber | T_2_ | 28 | N100Fc | 28 | 11 |
| 4 Septmber | T_2_ | 7 | N50Fa | 7 | 14 |
| 4 Septmber | T_2_ | 18 | N50Fb | 18 | 14 |
| 4 Septmber | T_2_ | 29 | N50Fc | 29 | 15 |
| 4 Septmber | T_2_ | 27 | N20Fa | 27 | 16 |
| 4 Septmber | T_2_ | 8 | N20Fb | 8 | 17 |
| 4 Septmber | T_2_ | 10 | N20Fc | 10 | 15 |
| 4 Septmber | T_2_ | 23 | C1 | 23 | 11 |
| 4 Septmber | T_2_ | 26 | C2 | 26 | 12 |
| 4 Septmber | T_2_ | 34 | C3 | 34 | 11 |
| 28 Septmber | T_3_ | 25 | H100Ufa | 25 | 9 |
| 28 Septmber | T_3_ | 11 | H100Ufb | 11 | 10 |
| 28 Septmber | T_3_ | 4 | H100Ufc | 4 | 8 |
| 28 Septmber | T_3_ | 9 | H50Ufa | 9 | 7 |
| 28 Septmber | T_3_ | 30 | H50Ufb | 30 | 6 |
| 28 Septmber | T_3_ | 12 | H50Ufc | 12 | 7 |
| 28 Septmber | T_3_ | 20 | H20Ufa | 20 | 5 |
| 28 Septmber | T_3_ | 36 | H20Ufb | 36 | 6 |
| 28 Septmber | T_3_ | 21 | H20Ufc | 21 | 6 |
| 28 Septmber | T_3_ | 5 | H100Fa | 5 | 10 |
| 28 Septmber | T_3_ | 13 | H100Fb | 13 | 7 |
| 28 Septmber | T_3_ | 32 | H100Fc | 32 | 8 |
| 28 Septmber | T_3_ | 2 | H50Fa | 2 | 12 |
| 28 Septmber | T_3_ | 6 | H50Fb | 6 | 11 |
| 28 Septmber | T_3_ | 16 | H50Fc | 16 | 12 |
| 28 Septmber | T_3_ | 33 | H20Fa | 33 | 16 |
| 28 Septmber | T_3_ | 22 | H20Fb | 22 | 18 |
| 28 Septmber | T_3_ | 15 | H20Fc | 15 | 17 |
| 28 Septmber | T_3_ | 37 | N100Ufa | 37 | 13 |
| 28 Septmber | T_3_ | 17 | N100Ufb | 17 | 15 |
| 28 Septmber | T_3_ | 1 | N100Ufc | 1 | 14 |
| 28 Septmber | T_3_ | 14 | N50Ufa | 14 | 11 |
| 28 Septmber | T_3_ | 38 | N50Ufb | 38 | 12 |
| 28 Septmber | T_3_ | 35 | N50Ufc | 35 | 12 |
| 28 Septmber | T_3_ | 24 | N20Ufa | 24 | 10 |
| 28 Septmber | T_3_ | 31 | N20Ufb | 31 | 7 |
| 28 Septmber | T_3_ | 19 | N20Ufc | 19 | 8 |
| 28 Septmber | T_3_ | 3 | N100Fa | 3 | 6 |
| 28 Septmber | T_3_ | 39 | N100Fb | 39 | 7 |
| 28 Septmber | T_3_ | 28 | N100Fc | 28 | 6 |
| 28 Septmber | T_3_ | 7 | N50Fa | 7 | 9 |
| 28 Septmber | T_3_ | 18 | N50Fb | 18 | 9 |
| 28 Septmber | T_3_ | 29 | N50Fc | 29 | 10 |
| 28 Septmber | T_3_ | 27 | N20Fa | 27 | 11 |
| 28 Septmber | T_3_ | 8 | N20Fb | 8 | 12 |
| 28 Septmber | T_3_ | 10 | N20Fc | 10 | 10 |
| 28 Septmber | T_3_ | 23 | C1 | 23 | 6 |
| 28 Septmber | T_3_ | 26 | C2 | 26 | 7 |
| 28 Septmber | T_3_ | 34 | C3 | 34 | 6 |
