# Supplementary material for: Consumer Depletion Alters Seagrass Resistance to an Invasive Macroalga
Source: PLoS One. 2015 Feb 27;10(2):e0115858. doi: 10.1371/journal.pone.0115858 (PMC4344340; doi:10.1371/journal.pone.0115858)
Supplement: S2 Table — Scouring from leaf movement (Scour), water flow, and sedimentation (Sediment) values at all treatment combinations (treatment abbreviations considering: shoot density (100%, 50%, and 20%) and height (Natural N and halved H) in Fenced (F) and unfenced (UF) units). C: control units. (DOCX) [file pone.0115858.s002.docx]

| **ENVIRONMENTAL VARIABLES** | | | | | | |
| --- | --- | --- | --- | --- | --- | --- |
| **Date** | **Time** | **Unit** | **Treatment** | **Sediment (g)** | **Scour (%)** | **Water flow (cm s^-1^)** |
| 06-07 August | T_1_ | 25 | H100Ufa | 0,137 | 2,5 | 3,65 |
| 06-07 August | T_1_ | 11 | H100Ufb | 0,124 | 2 | 1,825 |
| 06-07 August | T_1_ | 4 | H100Ufc | 0,136 | 1,5 | 1,825 |
| 06-07 August | T_1_ | 9 | H50Ufa | 0,139 | 2 | 1,825 |
| 06-07 August | T_1_ | 30 | H50Ufb | 0,128 | 1,5 | 3,65 |
| 06-07 August | T_1_ | 12 | H50Ufc | 0,132 | 2 | 1,825 |
| 06-07 August | T_1_ | 20 | H20Ufa | 0,128 | 2 | 1,825 |
| 06-07 August | T_1_ | 36 | H20Ufb | 0,134 | 2 | 3,65 |
| 06-07 August | T_1_ | 21 | H20Ufc | 0,136 | 2 | 3,65 |
| 06-07 August | T_1_ | 5 | H100Fa | 0,129 | 1,5 | 1,825 |
| 06-07 August | T_1_ | 13 | H100Fb | 0,128 | 1,5 | 3,65 |
| 06-07 August | T_1_ | 32 | H100Fc | 0,139 | 1,5 | 1,825 |
| 06-07 August | T_1_ | 2 | H50Fa | 0,124 | 1,5 | 3,65 |
| 06-07 August | T_1_ | 6 | H50Fb | 0,125 | 2 | 1,825 |
| 06-07 August | T_1_ | 16 | H50Fc | 0,128 | 1,5 | 3,65 |
| 06-07 August | T_1_ | 33 | H20Fa | 0,126 | 2,5 | 1,825 |
| 06-07 August | T_1_ | 22 | H20Fb | 0,137 | 2,5 | 1,825 |
| 06-07 August | T_1_ | 15 | H20Fc | 0,127 | 2 | 3,65 |
| 06-07 August | T_1_ | 37 | N100Ufa | 0,130 | 2 | 3,65 |
| 06-07 August | T_1_ | 17 | N100Ufb | 0,131 | 2 | 1,825 |
| 06-07 August | T_1_ | 1 | N100Ufc | 0,131 | 2 | 3,65 |
| 06-07 August | T_1_ | 14 | N50Ufa | 0,132 | 2 | 3,65 |
| 06-07 August | T_1_ | 38 | N50Ufb | 0,138 | 2 | 1,825 |
| 06-07 August | T_1_ | 35 | N50Ufc | 0,129 | 2,5 | 1,825 |
| 06-07 August | T_1_ | 24 | N20Ufa | 0,123 | 2,5 | 3,65 |
| 06-07 August | T_1_ | 31 | N20Ufb | 0,129 | 2,5 | 1,825 |
| 06-07 August | T_1_ | 19 | N20Ufc | 0,133 | 2 | 1,825 |
| 06-07 August | T_1_ | 3 | N100Fa | 0,127 | 1,5 | 1,825 |
| 06-07 August | T_1_ | 39 | N100Fb | 0,135 | 1,5 | 1,825 |
| 06-07 August | T_1_ | 28 | N100Fc | 0,124 | 1,5 | 1,825 |
| 06-07 August | T_1_ | 7 | N50Fa | 0,139 | 5 | 3,65 |
| 06-07 August | T_1_ | 18 | N50Fb | 0,124 | 1,5 | 1,825 |
| 06-07 August | T_1_ | 29 | N50Fc | 0,130 | 2,5 | 1,825 |
| 06-07 August | T_1_ | 27 | N20Fa | 0,133 | 2,5 | 3,65 |
| 06-07 August | T_1_ | 8 | N20Fb | 0,125 | 2 | 3,65 |
| 06-07 August | T_1_ | 10 | N20Fc | 0,134 | 1,5 | 3,65 |
| 06-07 August | T_1_ | 23 | C1 | 0,126 | 2,5 | 3,65 |
| 06-07 August | T_1_ | 26 | C2 | 0,129 | 2 | 1,825 |
| 06-07 August | T_1_ | 34 | C3 | 0,128 | 2 | 1,825 |
| 06-07 August | T_2_ | 25 | H100Ufa | 1,244 | 5 | 9,125 |
| 26-27 August | T_2_ | 11 | H100Ufb | 1,239 | 6 | 10,95 |
| 26-27 August | T_2_ | 4 | H100Ufc | 1,241 | 5,5 | 9,125 |
| 26-27 August | T_2_ | 9 | H50Ufa | 1,236 | 5,5 | 9,125 |
| 26-27 August | T_2_ | 30 | H50Ufb | 1,247 | 6,5 | 9,125 |
| 26-27 August | T_2_ | 12 | H50Ufc | 1,235 | 5,5 | 10,95 |
| 26-27 August | T_2_ | 20 | H20Ufa | 1,239 | 6,5 | 10,95 |
| 26-27 August | T_2_ | 36 | H20Ufb | 1,241 | 6,5 | 9,125 |
| 26-27 August | T_2_ | 21 | H20Ufc | 1,244 | 6,5 | 9,125 |
| 26-27 August | T_2_ | 5 | H100Fa | 1,234 | 5 | 9,125 |
| 26-27 August | T_2_ | 13 | H100Fb | 1,245 | 5 | 9,125 |
| 26-27 August | T_2_ | 32 | H100Fc | 1,250 | 5,5 | 10,95 |
| 26-27 August | T_2_ | 2 | H50Fa | 1,233 | 6 | 10,95 |
| 26-27 August | T_2_ | 6 | H50Fb | 1,236 | 6 | 10,95 |
| 26-27 August | T_2_ | 16 | H50Fc | 1,236 | 5,5 | 9,125 |
| 26-27 August | T_2_ | 33 | H20Fa | 1,237 | 5 | 10,95 |
| 26-27 August | T_2_ | 22 | H20Fb | 1,241 | 5 | 10,95 |
| 26-27 August | T_2_ | 15 | H20Fc | 1,236 | 5 | 9,125 |
| 26-27 August | T_2_ | 37 | N100Ufa | 1,242 | 6 | 9,125 |
| 26-27 August | T_2_ | 17 | N100Ufb | 1,244 | 6,5 | 9,125 |
| 26-27 August | T_2_ | 1 | N100Ufc | 1,241 | 5 | 9,125 |
| 26-27 August | T_2_ | 14 | N50Ufa | 1,243 | 5,5 | 10,95 |
| 26-27 August | T_2_ | 38 | N50Ufb | 1,236 | 6 | 9,125 |
| 26-27 August | T_2_ | 35 | N50Ufc | 1,234 | 6 | 9,125 |
| 26-27 August | T_2_ | 24 | N20Ufa | 1,237 | 6 | 9,125 |
| 26-27 August | T_2_ | 31 | N20Ufb | 1,247 | 5,5 | 9,125 |
| 26-27 August | T_2_ | 19 | N20Ufc | 1,241 | 5,5 | 10,95 |
| 26-27 August | T_2_ | 3 | N100Fa | 1,242 | 5,5 | 9,125 |
| 26-27 August | T_2_ | 39 | N100Fb | 1,238 | 6 | 9,125 |
| 26-27 August | T_2_ | 28 | N100Fc | 1,242 | 6 | 9,125 |
| 26-27 August | T_2_ | 7 | N50Fa | 1,239 | 6 | 10,95 |
| 26-27 August | T_2_ | 18 | N50Fb | 1,245 | 6 | 10,95 |
| 26-27 August | T_2_ | 29 | N50Fc | 1,231 | 6 | 10,95 |
| 26-27 August | T_2_ | 27 | N20Fa | 1,234 | 5,5 | 9,125 |
| 26-27 August | T_2_ | 8 | N20Fb | 1,235 | 5,5 | 9,125 |
| 26-27 August | T_2_ | 10 | N20Fc | 1,247 | 5,5 | 9,125 |
| 26-27 August | T_2_ | 23 | C1 | 1,245 | 6 | 10,95 |
| 26-27 August | T_2_ | 26 | C2 | 1,239 | 6,5 | 9,125 |
| 26-27 August | T_2_ | 34 | C3 | 1,251 | 6,5 | 9,125 |
